# The evolution of irreversible cell differentiation under cell death effect

**DOI:** 10.1371/journal.pone.0315255

**Published:** 2025-08-07

**Authors:** Yuanxiao Gao, Xueyan Zhao, Caixia Li

**Affiliations:** School of Mathematics and Data Science, Shaanxi University of Science and Technology, Xi’an, Shaanxi, China; Instituto Nacional de Medicina Genomica, MEXICO

## Abstract

Cell differentiation is an important characteristic of multicellular organisms which produce new-typed cells to engage in diverse life functions. Irreversible differentiation, as an important differentiation pattern, describes cells differentiated by determined trajectories to form specialized cell types. It has been found that differentiated cell types often show different death rates. Yet, it is still unclear what role cell death plays in shaping the formation of irreversible cell differentiation. Here, we establish a theoretical model to investigate the impact of cell death on the evolution of irreversible cell differentiation in multicellular organisms. Irreversible differentiation refers to the loss of a cell type’s differentiation potential, and it is constructed by the sequences of differentiation probabilities of a cell type across cell divisions. We show that irreversible differentiation is more likely to occur when cell death rates between cell types are linear. Meanwhile, differences in death rates between cell types affect the emergence conditions of irreversible differentiation, whereas no significant impacts on that from equal cell death rates. Additionally, we found that cell death impacts the cell number and cell composition of a mature organism. These findings provide insights into understanding the role of cell death in the formation of cells’ irreversible differentiation.

## Introduction

Cell differentiation – the developmental process through which undifferentiated cells acquire specialized functions – serves as a defining hallmark of multicellular organisms [1–5]. Cell differentiation allows complex multicellular organisms to maintain varying life functions through different specialized cell types e.g., nerve cells, muscle cells, and other specialized cell types [6–8]. Many mechanisms have been introduced to explain cellular differentiation [4, 5, 9–12] as the emergence of new cell type is still unclear [8]. Along with newly formed cell types, the cell differentiation process shows different differentiation patterns in terms of the differentiation potential, such as irreversible differentiation and reversible differentiation [13–15]. The most striking example is germ-soma differentiation in which germ-line cells (reproductive cells) are responsible for genetic information transmission and somatic cells (body cells) execute physiological functions. The two cell types are both the result of an irreversible differentiation process where cells eventually lose their cell differentiation capability across cell divisions in an organism [16]. However, their cell fates are different. Germ-line cells can pass to the next generation and develop into a new organism, whereas non-reproductive somatic cells have a limited lifetime and eventually undergo senescence and cell death. Thus, cell differentiation produces different types of cells that undergo varying life spans in an organism’s development, depending on cell types.

Cell death is a complex biological process encompassing distinct types (e.g. apoptosis, necrosis, autophagy) mediated through diverse mechanisms [17–21]. In multicellular organisms, this phenomenon occurs not only when an individual’s death but also throughout an individual’s developmental processes. Even without external environmental stimuli, cells can still undergo cell death throughout organisms’ development known as programmed cell death (genetically regulated self-destruction mechanism) [18]. Cell death has various functions, including regulation of cell number, elimination of abnormal and dangerous cells, sculpting or deletion of structures [17, 18, 21]. Studies have shown that specific cell types can exhibit programmed death patterns. For example, in *C. elegans*, the programmed death of somatic cells (non-reproductive cells) is strictly controlled by cell lineage [22]. Meanwhile, abnormal regulation of programmed cell death in humans accounts for a wide range of diseases, including developmental disorders and cancer [18]. Cell differentiation and cell death, together with cell proliferation serve a crucial relationship in an individual’s development and tissue homeostasis [21]. Cell death is often a consequence of mistakes that occur among cell division and differentiation [18]. In turn, cell death can secrete factors that stimulate cell proliferation and differentiation [18].

Although cell differentiation and cell death have both been extensively investigated, few works have examined their relationship from an evolutionary perspective. Theoretical work of cell differentiation is mostly focusing on emergence of task allocation between cells in the first place under the division of labour hypothesis which is a evolutionary principle where cells specialize to enhance group efficiency [14, 23–34]. Theoretical models of irreversible patterns of cell differentiation have not been paid enough attention previously probably due to lacking a mathematical tool to describe different patterns together with the complexity and high dimensions in such models. Recently, irreversible differentiation has been investigated in multicellular organism models with two cell types via sequences of stochastic cell differentiation probabilities over cell divisions [14, 35]. Irreversibility of cell differentiation was investigated in a model by considering the gene regulatory network [15]. However, cell death has not been considered in these models though previous studies have shown that natural selection favors cell death in clustered multicellularity as it can promote organisms’ reproduction [36, 37]. Meanwhile, research has also shown that dying cells can affect the cellular environment and trigger tissue regeneration [38]. Thus, the investigation between cellular mortality and cell differentiation patterns will therefore provide critical insights into the stabilizing irreversible cell fate determination patterns.

In the work, we propose a theoretical model to study the impact of cell death on the evolution of irreversible cell differentiation based on previous theoretical research [35]. Here, the cell differentiation probability can change depending on its division state [35]. Cell differentiation patterns are constructed by the sequences of differentiation probabilities across cell divisions. Irreversible differentiation refers to the differentiation probability sequences of a cell type converges to 0. We considered two functionally different cell types: germ-like and soma-like. Cell death is accompanied by cell division and differentiation in an organism. We adopt an organism’s reproductive rate as a proxy of an organism’s fitness, i.e. the expected number of offspring of the organism. This study will investigate how cellular mortality levels influence the emergence of irreversible specialization in two distinct cell lineages. Our conclusions show that irreversible differentiation emerges mostly when cell death rates between cell types are linear. The emergence conditions of irreversible differentiation change with different cell death rates but have no significant change under the same cell death rates. Finally, we found that cell death affects the cell number and cell composition of mature organisms.

## Model and methods

### Life cycle

**Fig 1:**
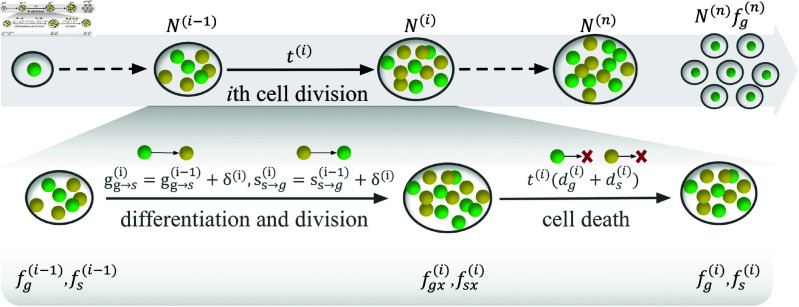
Schematic of the life cycle of an organism with cell death. The green spheres represent germ-like cells, and the yellow spheres represent soma-like cells. An organism is born with a germ-like cell, and cells do synchronous cell divisions with differentiation and cell death until *n* rounds of cell divisions. Then, the germ-like cells are released as offspring. We assume that cells die instantaneously after cell divisions and differentiation. ${\text{N}}^{\left(\text{i}\right)}$ is the cell number after the *i*th cell division. $f_{gx}^{\left(i\right)}$ ($f_{sx}^{\left(i\right)}$) is the proportion of germ-like cells (soma-like cells) after the *i*th cell differentiation. $f_{g}^{\left(i\right)}$ ($f_{s}^{\left(i\right)}$) is the proportion of germ-like cells (soma-like cells) after the *i*th cell death. ${\text{t}}^{\left(\text{i}\right)}$ is the waiting time from the (*i*–1)th cell division to the *i*th cell division, see the main text for more detail.

In the model, we consider multicellular organisms that can have two cell types (Fig 1). This model set is based on previous work where the cell type setting is inspired by *Volovx*, two functionally different cell types are considered: germ-like and soma-like [14, 35]. Germ-like cells are responsible for reproduction whereas soma-like cells are responsible for survival. We should note that the two cell types are the transient cell types rather than the final determined cell types. Thus cells can differentiate to another cell type via cell division. Each organism is born with a germ-like cell and then undergoes division with differentiation and death. Then each cell undergoes synchronous division producing two daughter cells until the organism matures after *n*th cell divisions which is a given value of interest. After maturity, all soma-like cells die and germ-like cells turn into new offspring organisms and repeat the life cycle. During cells’ division, they can differentiate with certain probabilities. For instance, there are three division pathways for germ-like cells: divide into two germ-like cells with a probability of *g*_*gg*_, differentiation into one germ-like cell, and divide one soma-like cell with a probability of *g*_*gs*_, differentiation into two soma-like cells with a probability of *g*_*ss*_. Here, $g_{gg}\;+\;g_{gs}\;+\;g_{ss}=1$. Differentiation probabilities can also be expressed as $g_{g\to s}^{\left(i\right)}$ which is $g_{ss}^{\left(i\right)}\;+\;\frac{1}{2}g_{gs}^{\left(i\right)}$, where *i* represents the *i*th cell division. The probabilities between two adjacent cell divisions are randomly taken but with a difference i.e. $g_{g\to s}^{i}=g_{g\to s}^{\left(i-1\right)}\;+\;\delta^{\left(i\right)}$, where $|\delta^{\left(i\right)}|\leq 1$. Soma-like cells divide similarly to germ-like cells.

### Differentiation strategy

Differentiation strategy is defined by the sets of sequences of differentiation probabilities of cell types. In the sequence, the first element consists of six probabilities $g_{gg}^{\left(1\right)}$, $g_{gs}^{\left(1\right)}$, $g_{ss}^{\left(1\right)}$, $s_{gg}^{\left(1\right)}$, $s_{gs}^{\left(1\right)}$, and $s_{ss}^{\left(1\right)}$ which are randomly chosen to execute cell differentiation. Then, the cell differentiation probabilities for the second cell division are deviated at most $\delta^{\left(1\right)}$. Similar methods for taking the following differentiation probabilities until the organism reaches maturity. We take the same definition of cell differentiation strategy as Gao’s work [35]. Cell differentiation patterns include non-differentiation (*ND*), reversible differentiation (*RD*), and irreversible differentiation (*ID*) based on the differentiation probabilities at the last cell division. Among them, non-differentiation (*ND*) means that cells do not differentiate and only proliferate germ-like cells, i.e. $g_{gg}^{\left(i\right)}=1$ for i=1,2,…,n. Irreversible differentiation (*ID*) indicates the presence of germ-like cell differentiation in the first *n*–1 divisions i.e. there exists at least once $g_{g\to s}^{\left(i\right)}<1$ for i=1,…,(n−1). At the last cell division i.e. *i* = *n*, either $g_{g\to s}^{\left(i\right)}=0$ or $s_{s\to g}^{\left(i\right)}=0$. Reversible differentiation (*RD*) is the rest strategy that with $g_{g\to s}^{\left(n\right)}>0$ and $s_{s\to g}^{\left(n\right)}>0$.

### Reproductive rate

We measure the performance of differentiation strategies by using an organism’s expected reproductive rate which is the number of germ-like cells after *n*th cell division [35]. The cell differentiation strategy which leads to an organism growing fastest will be selected and be denoted as optimal one. The reproductive rate of an organism is defined as:

$$\lambda =\frac{ln(N^{\left(n\right)}f_{g}^{\left(n\right)})}{t}\;=\frac{ln(N^{\left(n\right)}f_{g}^{\left(n\right)})}{\sum_{i=1}^{n}\frac{1}{r^{\left(i\right)}}}\;=\frac{ln(N^{\left(n\right)}f_{g}^{\left(n\right)})}{\sum_{i=1}^{n}\frac{1+c(f_{g}^{\left(i-1\right)}g_{g\to s}^{\left(i\right)}+\alpha f_{s}^{\left(i-1\right)}s_{s\to g}^{\left(i\right)})}{1+bf_{s}^{\left(i-1\right)}}},$$ (1)

where ${\text{N}}^{\left(\text{n}\right)}$ is the total cell number and $f_{g}^{\left(n\right)}$ is the fraction of germ-like cells of the mature organism, respectively. ${\text{r}}^{\left(\text{i}\right)}$ is the cell division rate between the (*i*–1)th and the *i*th division, which is defined as $r^{\left(i\right)}=\frac{1+bf_{s}^{\left(i-1\right)}}{1+c(f_{g}^{\left(i-1\right)}g_{g\to s}^{\left(i\right)}+\alpha f_{s}^{\left(i-1\right)}s_{s\to g}^{\left(i\right)})}$. $t^{\left(i\right)}=\frac{1}{r^{\left(i\right)}}$ is the expected growth time between the (*i*–1)th and the *i*th division. Thus, $t=\sum_{i=1}^{n}t^{\left(i\right)}=\sum_{i=1}^{n}\frac{1}{r^{\left(i\right)}}$ is the expected growth time of an organism. *b*, *c* and *α* measure the effects of cell interactions. Specifically, *b* and *c* represent the benefits and costs that a differentiation strategy brings to an organism respectively. $f_{g}^{\left(i\right)}$ ($f_{s}^{\left(i\right)}$) represents the fraction of the germ-like (soma-like) cells after the *i*th cell division.

### Cell number under cell death

Next, to calculate an organism’s reproductive rate, we calculate $f_{g}^{\left(i\right)}$, $f_{s}^{\left(i\right)}$, and ${\text{N}}^{\left(\text{n}\right)}$ first. We assume that $d_{g}^{\left(i\right)}$ and $d_{s}^{\left(i\right)}$ are the death rates per unit time of germ-like cells and soma-like cells at *i*th cell division respectively i.e. during the time between the (*i*–1)th and the *i*th division. Hence, the cell death rate is proportional to the waiting time between adjacent cell divisions (Fig 1). We employ state-dependent cell death rates because cellular mortality can stem from a cascade of molecular processes triggered by diverse physiological or developmental cues [21]. For instance, lysosomal membrane permeabilization (LMP), a recognized cell death mechanism, can be mediated by temporally dynamic factors such as photodamage, DNA damage, toxin exposure, or other stress-inducing stimuli [39]. We assume that cells die instantaneously after cell divisions and differentiation as cell death is often a consequence of the mistakes that occur among cell division and differentiation [18]. Therefore, we first calculate the change in cell fractions caused by cell division and differentiation, and then cell death. For convenience, we use $f_{gx}^{\left(i\right)}$ ($f_{sx}^{\left(i\right)}$) to denote the cell fraction of germ-like cell (soma-like cell) after the *i*th cell differentiation, and $f_{g}^{\left(i\right)}$ ($f_{s}^{\left(i\right)}$) to denote the cell fraction that after the *i*th cell death. The introduction of $f_{gx}^{\left(i\right)}$ and $f_{sx}^{\left(i\right)}$ serve to derive $f_{g}^{\left(i\right)}$ and $f_{s}^{\left(i\right)}$, which subsequently enable the quantification of distinct cell types during cell division events. The parameters $f_{gx}^{\left(i\right)}$ and $f_{sx}^{\left(i\right)}$ are first computed through iterative application of cell differentiation probabilities, while $f_{g}^{\left(i\right)}$ and $f_{s}^{\left(i\right)}$ are subsequently determined by integrating cell mortality rates into the established framework. Thus, we have

$$f_{gx}^{\left(i\right)}=f_{g\to g}^{\left(i\right)}+f_{s\to g}^{\left(i\right)}=f_{g}^{\left(i-1\right)}g_{g\to g}^{\left(i\right)}+f_{s}^{\left(i-1\right)}s_{s\to g}^{\left(i\right)}\;f_{sx}^{\left(i\right)}=f_{g\to s}^{\left(i\right)}+f_{s\to s}^{\left(i\right)}=f_{g}^{\left(i-1\right)}g_{g\to s}^{\left(i\right)}+f_{s}^{\left(i-1\right)}s_{s\to s}^{\left(i\right)}\;f_{g}^{\left(i\right)}=\frac{f_{gx}^{\left(i\right)}(1-d_{g}^{\left(i\right)}t^{\left(i\right)})}{f_{gx}^{\left(i\right)}(1-d_{g}^{\left(i\right)}t^{\left(i\right)})+f_{sx}^{\left(i\right)}(1-d_{s}^{\left(i\right)}t^{\left(i\right)})}\;f_{s}^{\left(i\right)}=\frac{f_{sx}^{\left(i\right)}(1-d_{s}^{\left(i\right)}t^{\left(i\right)})}{f_{gx}^{\left(i\right)}(1-d_{g}^{\left(i\right)}t^{\left(i\right)})+f_{sx}^{\left(i\right)}(1-d_{s}^{\left(i\right)}t^{\left(i\right)})},$$ (2)

where $f_{s\to g}^{\left(i\right)}$ is the fraction of soma-like cells that differentiate into the germ-like cell. ${\text{t}}^{\left(\text{i}\right)}$ is the expected time between (*i*–1)th and *i*th cell division, which is $\frac{1}{r^{\left(i\right)}}$. $g_{g\to s}^{\left(i\right)}$ refers to the probability of germ-like cells differentiating into soma-like cells during the *i*th division.

Next, we calculate the total cell number ${\text{N}}^{\left(\text{n}\right)}$. With cell death, the maturity organism size ${\text{N}}^{\left(\text{n}\right)}$ is smaller than $2^{\text{n}}$. Specifically, when the unit death rates of germ-like cells and soma-like cells are the same ($d_{g}^{\left(i\right)}=d_{s}^{\left(i\right)}$), the total number of mature cells ${\text{N}}^{\left(\text{n}\right)}$ is

$$N^{\left(n\right)}=N^{\left(0\right)}2^{n}\prod_{i=1}^{n}(1-d_{g}^{\left(i\right)}t^{\left(i\right)})=2^{n}\prod_{i=1}^{n}(1-d_{g}^{\left(i\right)}t^{\left(i\right)}),$$ (3)

where $t^{\left(i\right)}=\frac{1}{r^{\left(i\right)}}$. ${\text{N}}^{\left(0\right)}$ refers to the number of cells for the newborn organism, where there is only one germ-like cell, so *N*^(0)^ = 1. When the unit mortality rates of germ-like cells and soma-like cells are different ($d_{g}^{\left(i\right)}\neq d_{s}^{\left(i\right)}$), the total number of cells ${\text{N}}^{\left(\text{n}\right)}$ at the time of cell development and maturation is

$$N^{\left(n\right)}=N^{\left(0\right)}2^{n}\prod_{i=1}^{n}[1-t^{\left(i\right)}(f_{gx}^{\left(i\right)}d_{g}^{\left(i\right)}+f_{sx}^{\left(i\right)}d_{s}^{\left(i\right)})]\;=2^{n}\prod_{i=1}^{n}[1-t^{\left(i\right)}(f_{gx}^{\left(i\right)}d_{g}^{\left(i\right)}+f_{sx}^{\left(i\right)}d_{s}^{\left(i\right)})],$$ (4)

see S1 File for more detail.

## Results

To investigate the emergence conditions of irreversible differentiation, we numerically calculate the reproductive rates of organisms that adopt all potential differentiation strategies under varying parameters of cell death rates and differentiation benefits and costs, see S2 File for more detail. Different strategies compete via the reproductive rates of the organisms that the strategies acted on. Then we seek the optimal strategy that leads organisms to the highest reproductive rate through all parameter space. The parameter space where *ID* is optimal refers to the emergence conditions of irreversible differentiation. For simplicity, we assume that the cell death rate is stage-independent which means $d_{g}^{\left(i\right)}$ and $d_{s}^{\left(i\right)}$ are constant for different *i*. We denote them by *d*_*g*_ and *d*_*s*_ in the following investigation. For convenience, we set $|\delta^{\left(i\right)}|\leq 0.1$. The numerical calculation of the reproductive rate takes the same method as that in the previous study [35].

### Irreversible differentiation occurs mostly under linear death rates of different typed cells

**Fig 2:**
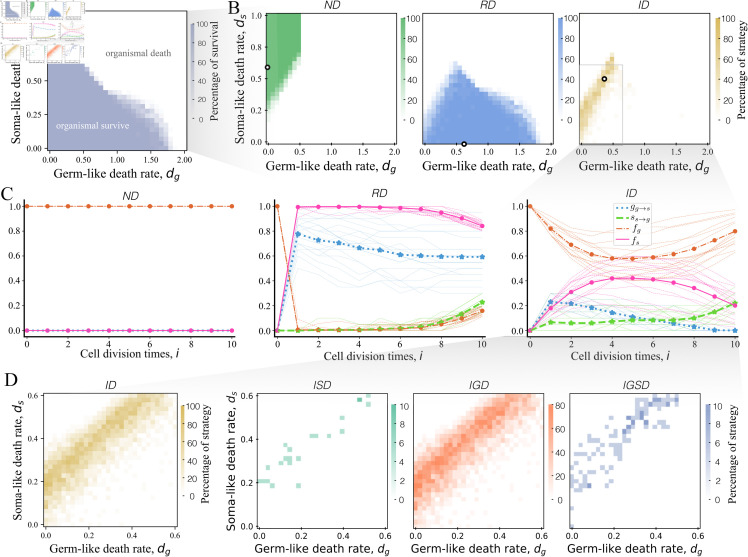
Optimal strategy under cell death rates. A: The survival space of organisms under cell death rates. The blue area represents the cell death rate space where cells can survive to mature. The white area indicates that organisms die by losing all their cells. B: The proportion of optimal strategy being *ND*, *RD* and *ID*, respectively. C: Cell differentiation probabilities ($g_{g\to s}$, $s_{s\to g}$) and the frequencies of cells (*f*_*g*_, *f*_*s*_) across cell divisions for the strategies that denoted by the white circles in panel *B*. D: The proportion of each optimal sub-strategy of *ID* under cell death rates, respectively. Parameters of all panels: maximal cell division times *n* = 10, $|\delta^{\left(i\right)}|\leq 0.1$, and *b* = *c* = *α* = 1. At each pixel, 20 replicates for calculating the optimal strategy.

A high cell death rate will lead to organismal death where no cells can survive to maturity. Therefore, we need to investigate the impact of cell death on organismal death before irreversible differentiation emerges. In the model, the cell death rates of germ-like cells and soma-like cells are *d*_*g*_ and *d*_*s*_ respectively. As each organism is born with a germ-like cell, thus high *d*_*g*_ will lead to the death of germ-like cells which further leads to organismal death. Thus, organisms cannot survive at high *d*_*g*_. Our numerical results show that when the cell death rate of germ-like cells increases to around 1.8, organisms die (Fig 2A). In comparison, a high soma-like cell death rate *d*_*s*_ alone will not lead to organismal death. Under this scenario, organisms that produce few soma-like cells will not die. An extreme example is the organisms with *ND* strategy, where the organism only contains germ-like cells, thus high *d*_*s*_ has no impact. Finally, a combined cell death rate of both cell types will lead to fast organismal death. We found that organisms is likely to die when *d*_*g*_ exceeds 0.5 and *d*_*s*_ exceeds 0.4 (Fig 2A). In the following investigation of differentiation strategies, we only consider the range of cell death rates that ensures organisms’ survival.

Our results show that irreversible differentiation strategy *ID* occurs in the parameter space between the space for strategy *ND* and *RD* emergence (Fig 2B). We found that *ID* evolves in low cell death rate both for germ-like cells *d*_*g*_ and soma-like cells *d*_*s*_. The growth of organisms with *ND* strategy is the clonal reproduction of germ-like cells and no soma-like cells (Fig 2C). Thus, *d*_*s*_ has no impact on *ND*. *ND* strategy can evolve in high *d*_*s*_. According to Eq 3, an organism can survive when its final cell number N≥1. That is

$$N=N^{\left(0\right)}2^{n}\prod_{i=1}^{n}(1-d_{g}^{\left(i\right)}t^{\left(i\right)})=2^{n}\prod_{i=1}^{n}(1-d_{g}^{\left(i\right)}t^{\left(i\right)})=2^{n}(1-d_{g}{)}^{n}\geq 1,$$ (5)

where ${\text{t}}^{\left(\text{i}\right)}$ equals 1 in *ND* according to Eq 1. Then, organisms that adopted strategy *ND* can survive when $d_{g}\leq 0.5$. Our numerical results are consistent with this conclusion, see Fig 2A and 2B. Strategies of *RD* evolve at high *d*_*g*_ as the strategy can keep relatively fewer germ-like cells in an organism to avoid cell death. Specifically, the optimal *RD* strategy exhibits an elevated differentiation propensity from germ-like to soma-like cells and a suppressed reverse differentiation rate until the terminal cell division cycles, as demonstrated in Fig 2C. The optimal strategy has the opposite cell differentiation probability tendency in the last few cell divisions as soma-like cells die at the end of the multicellular life cycle. Thus, the selected RD strategy can evolve by avoiding early germ-like cell loss while generating abundant germ-like cells at maturity, thereby boosting reproductive success. Since the fraction of germ-like cells produced by *ID* can be between that by strategy *ND* and strategy *RD*, *ID* emerges in the parameter space between the space that emerges for *ND* and *RD* (Fig 2C). Therefore, *ID* is optimal under selection of both *d*_*g*_ and *d*_*s*_.

We further found that *ID* emerges predominantly when *d*_*g*_ and *d*_*s*_ fall in a narrow strip space showing a linear relationship, see Fig 2B and 2D. Even though *ID* can emerge in these narrow strip areas where *d*_*g*_ and *d*_*s*_ do not necessarily show a strictly linear relationship. Nevertheless, the linear condition leads to the highest percentages of *ID* (Fig 2B and 2D). Notably, the conclusion does not exhibit a significant change when differentiation benefits *b* and costs *c* vary, see S3 File. This conclusion indicates that the two-typed cells need to keep proportional changes in cell death rate for the emergence of irreversible differentiation. The reason is that if there is a big difference between the two cell death rates, then either *ND* or *RD* will be selected. Specially, if the increase of *d*_*g*_ is larger than that of *d*_*s*_, then the strategy *RD* will be chosen. Since *RD* includes the strategy that produces a high fraction of soma-like cells in the early stages of cell division and as many germ-like cells as offspring in the late cell divisions. In contrast, if the increase of *d*_*s*_ is higher than that of *d*_*g*_, the strategy *ND* will be chosen as it only contains germ-like cells and thus can avoid cell death.

Furthermore, we investigate what kind of strategy is chosen in *ID*. Based on the definition, we know that at least one cell type has no cell differentiation in *ID*. Thus, *ID* includes three sub-strategies *IGD*, *ISD*, and *IGSD*, which are according to $g_{g\to s}^{\left(n\right)}=0$, $s_{s\to g}^{\left(n\right)}$ and $g_{g\to s}^{\left(n\right)}=s_{s\to g}^{\left(n\right)}=0$ respectively. The three sub-strategies together make strategy *ID* impacted by *d*_*g*_ and *d*_*s*_ simultaneously. We found that *IGD* is the most occurring sub-strategy in irreversible differentiation, see Fig 2D. The finding is consistent with previous study [35].

### Cell death rate differences between different cell types affect the emergence condition of irreversible differentiation

**Fig 3:**
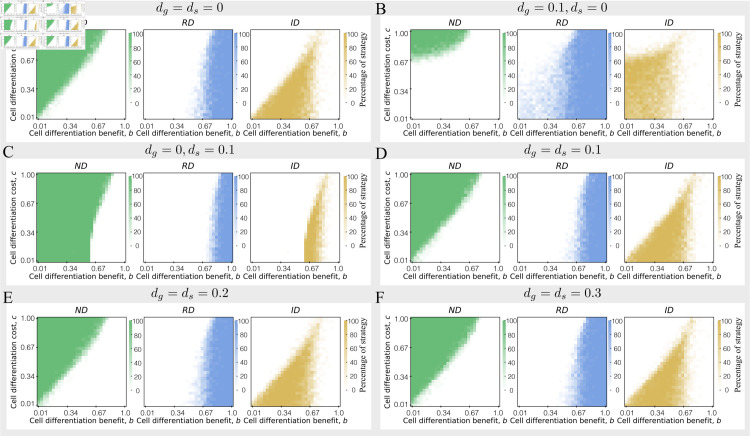
The percentage of optimal differentiation strategy under differentiation benefits and costs with varying cell death rates. The percentage of optimal differentiation strategy being *ND*, *RD*, and *ID* under differentiation benefits and differentiation costs in the condition of $d_{g}^{\left(i\right)}=d_{s}^{\left(i\right)}=0$ (panel A), $d_{g}^{\left(i\right)}=0.1$, $d_{s}^{\left(i\right)}=0$ (panel B), $d_{g}^{\left(i\right)}=0$, $d_{s}^{\left(i\right)}=0.1$ (panel C), $d_{g}^{\left(i\right)}=d_{s}^{\left(i\right)}=0.1$ (panel D), $d_{g}^{\left(i\right)}=d_{s}^{\left(i\right)}=0.2$ (panel E), $d_{g}^{\left(i\right)}=d_{s}^{\left(i\right)}=0.3$ (panel F). When the cell death rates of germ-like cells and soma-like cells are equal, although the cell death rate increases, its impact on the optimal strategy is not significant. Other parameter: *n* = 10, $|\delta^{\left(i\right)}|\leq 0.1$, α=1. At each pixel, 20 replicates for calculating the optimal strategy.

In this section, we investigate the impacts of cell death on the emergence condition of irreversible differentiation under differentiation benefits *b* and costs *c*. An organism will grow fast by producing soma-like cells which serve survival functions. Differentiation benefits measure the contribution of soma-like cells in an organism. The contribution will increase cell division rates and thus decrease an organism’s growth time [35]. Differentiation costs inhibit cell differentiation and punish cell differentiation by decreasing an organism’s reproductive rate, see Eq 1. Our analysis revealed that differential cell death rates significantly alter the evolutionary dynamics of irreversible differentiation, whereas equal rates result in no observable changes to these conditions, see Fig 3.

Without cell death, high differentiation costs prohibit cell differentiation, thus *ND* emerges at the area of high *c* and low *b*, see Fig 3A. Comparatively, when the differentiation benefits are large enough, differentiation strategy *RD* will be the optimal strategy. *ID* emerges in the parameter space with a certain amount of differentiation benefits and low differentiation costs. The conclusion is consistent with previous work [14, 35]. When germ-like cell death rate increases, differentiation strategies of *ID* and *RD* replace *ND* being the optimal one in the area with low differentiation benefits and low differentiation costs, see Fig 3B. This is because organisms with *ND* only have germ-like cells and thus have more cell death compared with organisms that adopted the other two strategies under the scenario of *d*_*g*_ but without *d*_*s*_. In contrast, when soma-like cell death increases, *ND* invades the parameter space which is occupied by *ID* and *RD*. As differentiation strategies (*RD* and *ID*) contain soma-like cells, they are more sensitive to soma-like cell death. Our results show that the evolving conditions of *ID* change differently by increasing the same amount of death rate for the two cell types. *ID* is likely to be found in the organism with germ-like cell death rather than soma-like cell death (Fig 3B and 3C).

However, most interestingly, we found that the conditions for evolving *ID* are unchanged much when both cell types possess the same death rate, Fig 3A, 3D–3F. The comparable death rate for both cell types will equally decrease the number of both cells but does not change their proportions. Thus, the proximate death rate impacts organisms similarly and eventually reduces an organism’s cell number at the maturity stage. However, it does not impact the evolving condition for different differentiation strategies. The result indicates that the same cell death rate for different cell types will not change the emergence conditions of the differentiation patterns. Therefore, the evolution of more complex and hierarchical structures needs distinct cell death rates between cell types. This discovery elucidates a critical regulatory mechanism by which cell death governs the establishment of cellular differentiation patterns.

### Cell death impacts organisms’ size and cell composition

**Fig 4:**
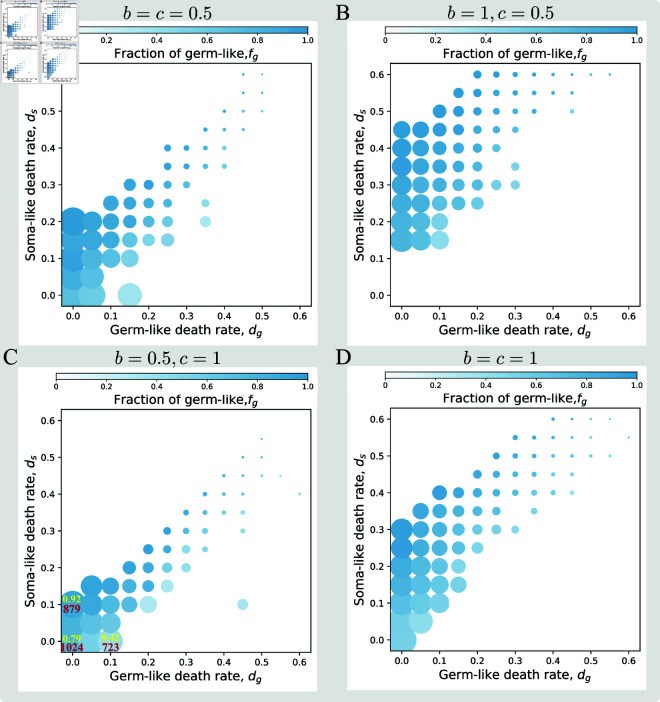
Cell death rates decrease cell number and affect cell fraction in organisms with *ID.* The averaged cell number and cell proportion of the organisms with *ID* strategy under *b* = 0.5, *c* = 0.5 in panel A, *b* = 1, *c* = 0.5 in panel B, *b* = 0.5, *c* = 1 in panel C and *b* = 1, *c* = 1 in panel D. The bubble size represents the average cell number of the maturity organisms with *ID* strategy. The bubble color represents the averaged proportion of germ-like cells within the organisms with *ID* strategy. The red and yellow numbers in panel C represent the specific values of the total number of cells and the proportion of germ-like cells, respectively. Parameters: *n* = 10, α=1. At each pixel, 10 replicates for calculating the optimal strategy.

Finally, we investigate the impact of cell death on organismal size and cell composition in mature organisms which adopted *ID* as the optimal strategy. Here, the organismal size is the total cell number that an organism can reach after *n* rounds of cell divisions. The cell composition is investigated by the fraction of germ-like cells. We found that organismal size decreases with both cell death rates *d*_*g*_ and *d*_*s*_, see Fig 4. Meanwhile, the proportion of a cell type is inversely proportional to the cell death rate of this type. Without cell death, cells undergo binary synchronous division, and an organism will eventually have $2^{\text{n}}$ cells. Therefore, we investigate the changes in organismal size and cell composition for the organism adopted *ID* by comparing them to those that are without cell death. Our numerical results show that the organismal size can be smaller to 1 under both *d*_*g*_ and *d*_*s*_ compared with 1024 cells without cell death for *n* = 10 (Fig 4C). Without cell death, the proportion of germ-like cells is around 79% which means 21% soma-like cells, see Fig 4C. When *d*_*g*_ increases to 0.1, the maturity organismal size decreases from 1024 to 723, and the proportion of germ-like cells decreases from 0.79 to 0.42. However, when *d*_*s*_ increases to 0.1, the maturity organismal size decreases from 1024 to 879, and the proportion of germ-like cells increases from 0.79 to 0.92. Comparatively, the amount of organism size decreases more than two folds for *d*_*g*_ decrease than that for *d*_*s*_ decrease, and the amount of the fraction of germ-like cells decreases more than three folds. These results demonstrate that the death of germ-like cells plays a more crucial role in influencing cell number and cell composition.

## Discussion

In the model, we assumed the two cell types are based on their life functions as the precise definition of cell type is still lacking, instead functional and morphological differences is viewed as characteristics of distinct cells [5, 40]. The two cell types are more like general cells but with different task preferences rather than specialized cell types. In other words, we investigated the cells that have functional preferences but are still in the state before finally turning into specialized cells by irreversible differentiation. These transient cell types allow us to investigate the cell differentiation patterns which need to screen the differentiation probabilities along with cell divisions. A similar way of defining cell types by cells’ function in other theoretical investigations of cell differentiation [13, 23, 27, 28, 34, 41]. An alternative way of cell types definition is the Boolean model, which is a discrete dynamical system comprising a set of Boolean variables (e.g., True/False, 0/1), the values depending on a set of functions applied to each variable [5]. Another method of cell types definition is via the fixed point attractors of a continuous dynamical system which is controlled by the expression levels of related genes [15].

Our framework distinguishes itself from prior research in cell differentiation through three aspects. First, we incorporate cellular mortality as a primary dynamic variable - a critical yet understudied factor in differentiation analysis [14, 23, 25, 27, 28, 31, 33, 34, 41]. This oversight in existing literature likely stems from the field’s focus on initial differentiation between cells, rather than their developmental trajectories. Initial differentiation states may only have minimal mortality variations and thus exert negligible influence on cell differentiation outcomes. Second, our model introduces a novel paradigm for cellular trajectory regulation, explicitly accounting for differentiation pathway plasticity rather than assuming predetermined fate commitments. Differentiation patterns of irreversible and reversible have been considered in filament-shaped organisms with two indispensable cell types [13]. Comparatively, our model contains organisms born with single germ cells and meanwhile can include the strategy of no differentiation strategy. Finally, our model adopts the reproductive rate as an organism’s fitness. To quantify cell differentiation, many models adopted the product of the tasks undertaken by different cells [23, 27, 28, 34, 41]. However, we use an organism’s reproductive rate as a fitness proxy to evaluate differentiation strategies. One of the reasons is that cell number and composition continuously change with cell death. Thus, the product of the tasks split by cells is challenging to quantify as it continuously changes. Another reason is that differentiation patterns like irreversible differentiation need to consider cells’ differentiation probabilities that are accompanied by cell division series. Thus, we took the final reproductive rate as a fitness proxy.

Additionally, we investigated the irreversibility of cells under 10 rounds of cell division. This is because the model-inspired organisms *Volvox* contains thousands of cells in total [16]. Thus, when *n* = 10, an organism will grow to contain 1024 cells in total without cell death. Altogether, our model provides an analytical method investigating the effects of cell death between cell types on organisms’ differentiation patterns. In the model, the cell death rate is designed as a stage-dependent variable $d_{g}^{\left(i\right)}$ and $d_{s}^{\left(i\right)}$. Although we take constant cell death rates *d*_*g*_ and *d*_*s*_ for convenience, cell death can be designed as a pair of functions depending on the factors causing cell death. For example, cell death is highly likely for some cells rather than others under the same environmental stimulus [36]. While our model offers valuable theoretical insights into understanding the effects of cell death on cell differentiation, it contains inherent limitations. First, the binary cell-type assumption fundamentally restricts biological generalizability and constrains the framework for extrapolation to complex organisms with diversified cellular lineages where multi-type interactions govern differentiation dynamics. Second, the imposed synchronized cell division becomes biologically implausible as cellular numbers increase, contrasting with real systems governed by asynchronous divisions regulated through cell-cycle checkpoints and microenvironmental cues. Despite these constraints, the framework can be applied in low-complexity tissues with a few cell types. However, critical methodological revisions are warranted – particularly in redefining natural selection direction, as tissues lack reproductive capacity. Future iterations should replace reproduction rate metrics with tissue fitness proxies such as maintaining tissue homeostasis, metabolic efficiency, or apoptosis resistance.

## Conclusion

In the paper, we adopted a multicellular organism model to investigate the impact of cell death on the evolution of irreversible cell differentiation by considering cell interaction effects of differentiation benefits and costs. Irreversible differentiation describes the loss process of cell differentiation ability and is defined by 0 differentiation probability at the last cell division [35]. Our results show irreversible differentiation emergencies mostly when the cell death rates of germ-like cells and soma-like cells change in a linear relationship. The result indicates that the cell death difference between two closely related cell lineages shouldn’t be too large to evolve irreversible differentiation. It also suggests regulated cell death in an organism needs to keep a close cell death on different cell types. Then, we found that irreversible differentiation can occur in new parameter space by implementing different cell death rates on cell types. Specifically, our results show that irreversible differentiation extends to high differentiation costs areas when germ-like cells die (Fig 3). Meanwhile, the emergence condition of irreversible differentiation is not influenced by equal cell death rates. The result implies the factors that cause the same death in different cell types do not change the organism’s differentiation patterns. Finally, both cell death rates can reduce the cell number and the fraction of their cell types in mature organisms. However, we found that germ-like cell death has a stronger impact on reducing organismal size and cell fraction compared with soma-like cell death.

## Supporting information

S1 File Cell number of maturity organisms under cell death. (PDF)

S2 File Numerical calculation of searching for the optimal strategy. (PDF)

S3 File Irreversible differentiation emerges mostly under linear cell death rates of different cell types. (PDF)

S3 Fig *ID* emerges mostly when *d*_*g*_ and *d*_*s*_ show linear relationship. (PDF)
