# Supplementary material for: The evolution of irreversible cell differentiation under cell death effect
Source: PLoS One. 2025 Aug 7;20(8):e0315255. doi: 10.1371/journal.pone.0315255 (PMC12331116; doi:10.1371/journal.pone.0315255)
Supplement: S1 File — (PDF) [file pone.0315255.s001.pdf]

Supporting Information of “The evolution of irreversible cell differentiation under cell death effect”

Yuanxiao Gao[1*], Xueyan Zhao[1], Caixia Li[1]

1 School of Mathematics and Data Science, Shaanxi University of Science and Technology, Xi'an, Shaanxi, China

* yxgao@sust.edu.cn

S1 File. Cell number of maturity organisms under cell death. With cell death, the cell number of a maturity organism $N^{(n)}$ changes with varying cell death rates. In our study, we considered two types of cell death scenarios: (i) the unit cell death rates of germ-like and soma-like cells were the same at each division i.e. $d_s^{(i)} = d_g^{(i)}$, and (ii) the unit cell death rates of germ-like and soma-like cells were different at each division i.e. $d_s^{(i)} \neq d_g^{(i)}$. Next, provide a detailed description.

Cell number of a maturity organism $N^{(n)}$ under $d_s^{(i)} = d_g^{(i)}$. If the unit cell death rate of germ-like cells and soma-like cells is the same, then we assume $d_g^{(i)} = d_s^{(i)} = d^{(i)}$. Assuming the total number of cells after the ith division is $N^{(i)}$, the total number of cells after undergoing a synchronous division is $2N^{(i)}$, and the time consumed is $t^{(i+1)}$. During this period, the total number of dead cells was $2d^{(i)}N^{(i)}t^{(i+1)}$. So the total number of cells $N^{(i+1)}$ during the $i+1$st division is expressed as the difference between the total number of cell death not considered and the dead cells, and is denoted as:

$$\begin{aligned} N^{(i+1)} &= 2N^{(i)} - 2d^{(i)}N^{(i)}t^{(i+1)} \\ &= 2N^{(i)}(1 - d^{(i)}t^{(i+1)}). \end{aligned} \tag{1}$$

By recursion, the total number of cells $N^{(n)}$ during the last division is

$$\begin{aligned} N &= 2^n N^{(0)} \prod_{i=1}^{n} (1 - d^{(i)}t^{(i)}) \\ &= 2^n \prod_{i=1}^{n} (1 - d^{(i)}t^{(i)}), \end{aligned} \tag{2}$$

where $N^{(i)}$ represents the total number of cells after the ith division, $2d^{(i)}N^{(i)}t^{(i+1)}$ represents the total number of cells that died within the time $t^{(i+1)}$ spent on the $i+1$th division, and $N^{(0)}$ represents the total number of cells before the first division. Since cell division begins with a single germ-like cell, $N^{(0)} = 1$.

Cell number of a maturity organism $N^{(n)}$ under $d_s^{(i)} \neq d_g^{(i)}$. If the unit cell death rates of germ-like cells and soma-like cells are different, then the number of germ-like cells and soma-like cells are two independent quantities at each division. We assume that the total number of germ-like cells is $N_g^{(i)}$ and the total number of soma-like cells is $N_s^{(i)}$, where i represents the ith division and

$$N_g^{(i)} = N^{(i)} f_{gx}^{(i+1)} \tag{3a}$$

$$N_s^{(i)} = N^{(i)} f_{sx}^{(i+1)}, \tag{3b}$$

According to the calculation method of (i), we provide the total number of cells at the $(i+1)$th division $N^{(i+1)}$,

$$\begin{aligned} N^{(i+1)} &= 2N^{(i)} - 2N_g^{(i)} t^{(i+1)} d_g^{(i)} - 2N_s^{(i)} t^{(i+1)} d_s^{(i)} \\ &= 2N^{(i)} - 2N^{(i)} f_{gx}^{(i+1)} t^{(i+1)} d_g^{(i)} - 2N^{(i)} f_{sx}^{(i+1)} t^{(i+1)} d_s^{(i)} \\ &= 2N^{(i)} [1 - t^{(i+1)} (f_{gx}^{(i+1)} d_g^{(i)} + f_{sx}^{(i+1)} d_s^{(i)})]. \end{aligned} \tag{4}$$

By recursion, the total number of cells $N^{(n)}$ during the last division is

$$\begin{aligned} N &= 2^n N^{(0)} \prod_{i=1}^{n} [1 - t^{(i)} (f_{gx}^{(i)} d_g^{(i)} + f_{sx}^{(i+1)} d_s^{(i)})] \\ &= 2^n \prod_{i=1}^{n} [1 - t^{(i)} (f_{gx}^{(i)} d_g^{(i)} + f_{sx}^{(i+1)} d_s^{(i)})], \end{aligned} \tag{5}$$

where $f_{gx}^{(i)}$ represents the proportion of germ-like cells in the ith division, $2N_g^{(i)} t^{(i+1)} d_g^{(i)}$ represents the total number of all germ-like cells that died within the time $t^{(i+1)}$ spent on the $i+1$th division, and $2N_s^{(i)} t^{(i+1)} d_s^{(i)}$ represents the total number of all soma-like cells that died within the time $t^{(i+1)}$ spent on the $i+1$ division.
