# Supplementary material for: The evolution of irreversible cell differentiation under cell death effect
Source: PLoS One. 2025 Aug 7;20(8):e0315255. doi: 10.1371/journal.pone.0315255 (PMC12331116; doi:10.1371/journal.pone.0315255)
Supplement: S2 File — (PDF) [file pone.0315255.s002.pdf]

Supporting Information of “The evolution of irreversible cell differentiation under cell death effect”

Yuanxiao Gao[1*], Xueyan Zhao[1], Caixia Li[1]

1 School of Mathematics and Data Science, Shaanxi University of Science and Technology, Xi’an, Shaanxi, China

* yxgao@sust.edu.cn

S2 File. Numerical calculation of searching for the optimal strategy. *Differentiation strategy space.* We use the same numerical method to calculate an organism’s reproductive rate [1]. In the model, the differentiation strategies are infinite. To simplify, we use discrete differentiation probabilities to capture the effects of all differentiation strategies. We use grid search to find all possible differentiation strategies. Division probabilities $(g_{gg}^{(i)}, g_{gs}^{(i)}, g_{ss}^{(i)}, s_{gg}^{(i)}, s_{gs}^{(i)}, s_{ss}^{(i)})$ are confined in the values of $0, 0.1, 0.2, \ldots, 1$. Therefore, $|\delta^{(i)}|$ is confined in the values of 0, 0.05, or 0.1. Then, differentiation probabilities $g_{g\to s}^{(i)}$ and $s_{s\to g}^{(i)}$ are confined in the values of $0, 0.05, 0.1, \ldots, 1$. Then, we receive the first set of differentiation probabilities of a strategy $g_{gg}^{(1)}, g_{gs}^{(1)}, g_{ss}^{(1)}, s_{gg}^{(1)}, s_{gs}^{(1)}, s_{ss}^{(1)}$. Then, with a difference of 0.1 or 0 randomly generate the sets of differentiation probabilities for each cell division until n times. Even with the discrete differentiation space, there are also numerous differentiation strategies. For example, if $n = 10$, we have 4356 choice for the first cell division, then each of them will have at least (for boundary conditions) 5 sets and at most 13 sets of differentiation probabilities for the second cell division. We will have at least $4356 \times (2^5)^{10}$ strategies in the generated differentiation probability space. We cannot explore all the strategies and calculate their reproductive rates for comparison. Instead, we use the Monte-Carlo methods randomly to sample differentiation strategies and then calculate and compare the reproductive rates of organisms under these strategies.

Search for the optimal differentiation strategy. To find the optimal strategy at a fixed parameter space of cell death, benefit, and cost, we first randomly choose 100 number of the first cell differentiation probabilities in the 4356 strategy space, then for each of them, we randomly choose 10 strategies. Then there are 1000 strategies in total. Then we calculate the reproductive rates of these strategies and find the strategy with the largest reproductive rate. Considering the huge number of strategies, we run the above process several duplicates depending on the circumstances. Then we get the percentages of each classified strategy being optimal at the fixed parameter values of cell death, benefit, and cost. Finally, we repeat the above calculation across all possible parameter spaces in terms of cell death, benefit, and cost.

References

1. Yuanxiao Gao, Yuriy Pichugin, Arne Traulsen, and Román Zapién-Campos. Evolution of irreversible differentiation under stage-dependent cell differentiation. *Scientific Reports*, 15(1):7786, 2025.
