# Supplementary material for: The evolution of irreversible cell differentiation under cell death effect
Source: PLoS One. 2025 Aug 7;20(8):e0315255. doi: 10.1371/journal.pone.0315255 (PMC12331116; doi:10.1371/journal.pone.0315255)
Supplement: S3 File — (PDF) [file pone.0315255.s003.pdf]

Supporting Information of “The evolution of irreversible cell differentiation under cell death effect”

Yuanxiao Gao[1*], Xueyan Zhao[1], Caixia Li[1]

1 School of Mathematics and Data Science, Shaanxi University of Science and Technology, Xi’an, Shaanxi, China

* yxgao@sust.edu.cn

S3 File. Irreversible differentiation emerges mostly under linear cell death rates of different cell types. The results show that irreversible differentiation emerges mostly when d_g and d_s show a linear relationship under different cell interactive parameter spaces (S3 Figure). Irreversible differentiation emerges at a higher soma-like death rate when differentiation benefits increase. In comparison, irreversible differentiation decreases when differentiation costs increase.
