# Supplementary material for: The evolution of irreversible cell differentiation under cell death effect
Source: PLoS One. 2025 Aug 7;20(8):e0315255. doi: 10.1371/journal.pone.0315255 (PMC12331116; doi:10.1371/journal.pone.0315255)
Supplement: S3 Fig — (PDF) [file pone.0315255.s004.pdf]

Supporting Information of "The evolution of irreversible cell differentiation under cell death effect"

Yuanxiao Gao[1*], Xueyan Zhao[1], Caixia Li[1]

1 School of Mathematics and Data Science, Shaanxi University of Science and Technology, Xi'an, Shaanxi, China

* yxgao@sust.edu.cn

S3 Fig. **ID emerges mostly when d_g and d_s show linear relationship.** A: The percentages of ND, RD and ID being optimal under varying cell death rates with no differentiation benefits and costs i.e. $b = 0$, $c = 0$. B: The percentages of ND, RD and ID being optimal under varying cell death rates, differentiation benefits and costs. Other parameters: $n = 10$ and $\alpha = 1$. Replicates for searching optimal strategy at each dot is 10.
